# Awareness, knowledge, and motivations about lifespan, healthspan, and Healthy Longevity Medicine in the general population: the HEalthy LOngevity (HELO) conceptual framework

**DOI:** 10.1007/s11357-025-01562-4

**Published:** 2025-02-24

**Authors:** Belinda Wang, Anna Szücs, Elena Sandalova, E. J. Horberg, Paul A. O’Keefe, Louis Island, Hans J. Meij, Sonny Rosenthal, Andrea B. Maier

**Affiliations:** 1 NUS Academy for Healthy Longevity, Yong Loo Lin School of Medicine, National University of Singapore, Singapore, Singapore; 2 Healthy Longevity Translational Research Program, Yong Loo Lin School of Medicine, National University of Singapore, Singapore, Singapore; 3 Division of Family Medicine, Yong Loo Lin School of Medicine, National University of Singapore, Singapore, Singapore; 4 Department of Human Movement Sciences, @Ageamsterdam, Faculty of Behavioural and Movement Sciences, Amsterdam Movement Sciences, Vrije Universiteit Amsterdam, De Boelelaan 1105, 1081 HV Amsterdam, Netherlands; 5 Department of Management, University of Exeter Business School, Exeter, UK; 6 Department of Human Genetics, Amsterdam University Medical Centre, Amsterdam, Netherlands; 7 College of Integrative Studies, Singapore Management University, Singapore, Singapore

**Keywords:** Aging, Awareness, Healthspan, Lifespan, Longevity, Motivation

## Abstract

The global population is ageing and the gap between lifespan (total years lived) and healthspan (years lived free of diseases) is increasing. Healthy Longevity Medicine (HLM) is an approach to optimise health and healthspan, and it has substantial public health implications. Despite those implications, the understanding of public perspectives on this field is lacking. The HEalthy LOngevity (HELO) framework was developed through a literature review guided by expert discussions across disciplines to include evidence-based concepts of health-related decision-making, ageing, and HLM. The framework organises concepts into three components. The first two components, awareness and knowledge, explore public perception and understanding of the healthy longevity field, respectively. The third component, motivations, reflects factors underlying motivations towards healthy longevity. These include personality, current behaviours, personal values and beliefs, and health-related perceptions. The framework outlines the theoretical foundation to explore public knowledge and interest in healthy longevity. The framework will be refined based on findings from qualitative focus groups in Singapore and then applied to quantitative population surveys globally. These HELO initiatives aim to inform strategies for integrating HLM into public healthcare, promoting health and healthspan.

## Introduction

By 2030, one in six people globally will be aged 60 years or older [1]. While people are living longer, these additional years are often spent with chronic diseases such as diabetes, cardiovascular disease, and cancer [2]. The gap between lifespan (total years lived) and healthspan (years free from diseases) is approximately nine years worldwide [3]. This healthspan-lifespan gap is an economic burden as health spending continues to rise internationally, reaching US$ 9.8 trillion, or 10.3% of global gross domestic product, in 2021 [4].

To reduce the healthspan-lifespan gap, Healthy Longevity Medicine (HLM) aims to optimise health and healthspan by targeting the ageing processes across the lifespan [5]. By combining practices from biological and clinical science, HLM enables proactive primary and secondary prevention against chronic health conditions [6], making it a potential public health strategy. The rise of private and public HLM clinics and companies with consumer products directed at measuring or improving health and healthspan [7] reflects the growing attention and demand from consumers, industries, and policymakers. Whilst HLM could have profound implications for public health, it remains unclear which populations seek or avoid these clinic services and consumer products.

Attitudes towards extending lifespan amongst older individuals have been studied [8–10]; however, research on healthspan is limited. In a survey of 1000 Americans, 65% of the sample indicated their preference to live up to 85 years with sustained mental or physical youthfulness, whereas nearly 80% said they would want to live beyond 120 years with guaranteed health [11]. A McKinsey Health Institute survey across 21 countries reported consensus amongst older adults aged 55 years and above on the importance of life purpose, stress management, meaningful connections, and independence for overall health [12]. In Hevolution’s global survey of 4000 members of the public and 1000 healthy longevity healthcare professionals, around 70% of respondents expressed concerns about the ethical implications of longevity science, with two-thirds believing it could increase socio-economic inequalities [13]. These surveys underscore some of the complexities of public perceptions around health, well-being, and healthspan, as well as potential factors hindering engagement with HLM.

The present article introduces the HEalthy LOngevity (HELO) framework and the psychological concepts underlying public views towards lifespan, healthspan, and HLM. This framework provides a conceptual basis for further work on public perceptions of HLM, specifically, the operationalisation of the global HELO survey, a new measurement instrument for gauging public awareness, knowledge, and motivations about HLM.

## Method

### Developing the HELO framework

The HELO framework was developed through a review of the literature guided by expert discussions involving clinicians and researchers from diverse disciplines, including geroscience, medicine, psychiatry, social and behavioural psychology, and public health. Team members identified and discussed relevant concepts and evidence from their respective disciplines and the literature that were linked to views towards lifespan, healthspan, or HLM. Given the scarcity of research on healthspan, evidence from related topics, such as health decision-making and views towards ageing and medical advancement, were also considered. The concepts employed in the HELO framework were built on more general theories from social and behavioural psychology, which propose that human behaviour is, in part, influenced by cognitive evaluations of anticipated outcomes as reinforcement for behaviour [14], as well as by an interplay of extrinsic factors (e.g. external rewards or avoidance of negative consequences) and intrinsic factors (e.g. medical, and individual characteristics such as personal satisfaction and enjoyment) [15, 16].

Once the team reached a consensus on the most relevant concepts pertaining to lifespan, healthspan, and HLM, these concepts were organised into a conceptual framework, forming the basis for the subsequent development of the HELO population survey.

### Developing the HELO survey

The HELO framework guided the design of the HELO survey, a quantitative questionnaire-based population survey. Each concept from the framework was represented in the survey by a set of questions, derived from validated instruments where possible. For new questions, the research team followed an evidence-based approach to ensure the questions aligned with concepts and findings from the literature. Where necessary, modifications to questionnaire items were made to account for cultural contexts and/or conceptual considerations, ensuring that the survey items remained relevant, clear, and appropriate for the target population. The HELO survey would be deployed across the eight countries involved in the HELO consortium [17], with Singapore leading the initiative. For the deployment of the HELO survey in Singapore, face validation with diverse members of the local population helped ensure that the survey and its translations into the other national languages of Mandarin, Malay, and Tamil were understandable and culturally appropriate.

## Results

### HELO conceptual framework

**Fig. 1:**
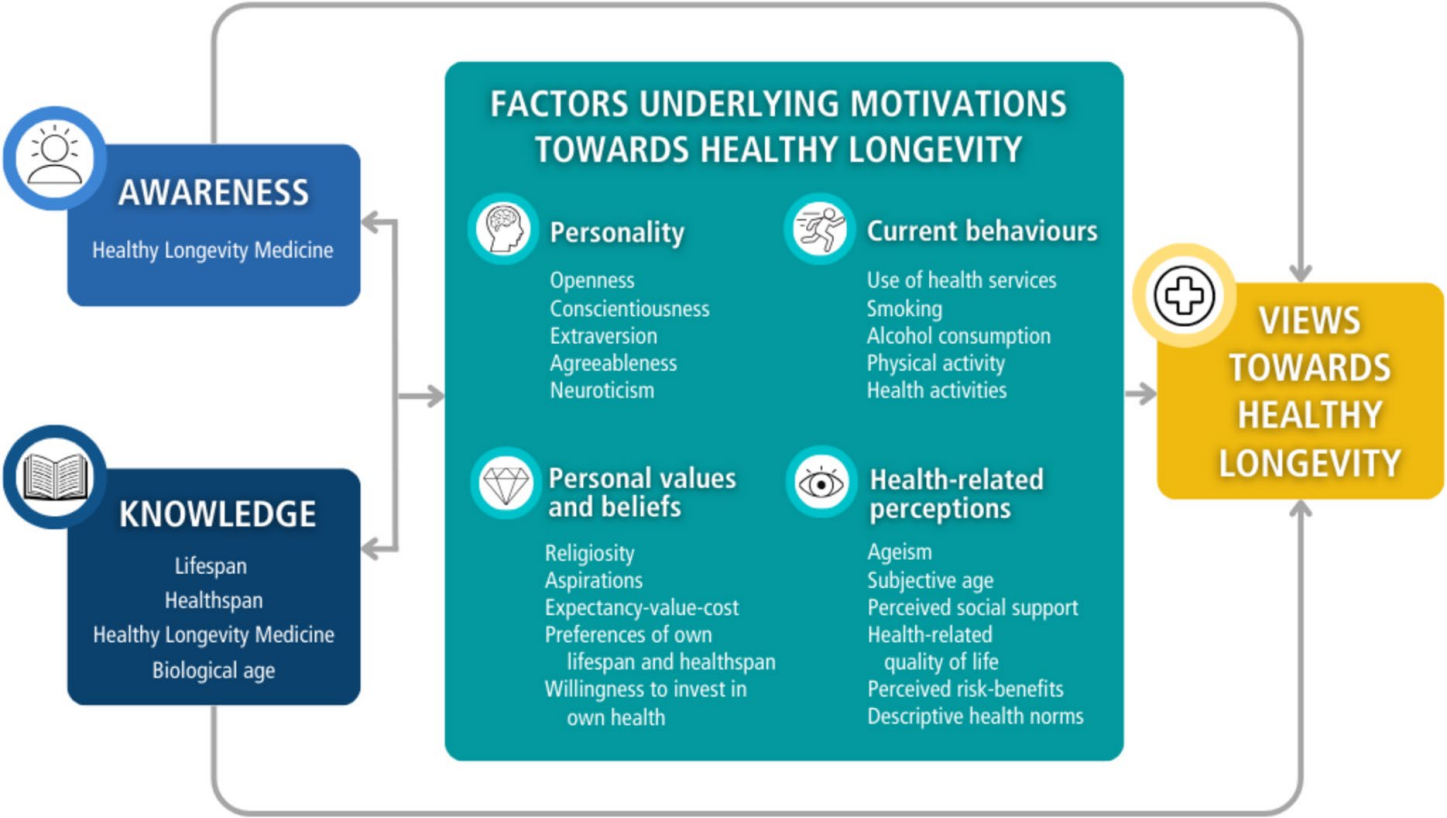
Conceptual framework of public awareness, knowledge, and factors underlying motivations that shape views towards healthy longevity

Identified concepts were organised into three components, which shape the views towards lifespan, healthspan, and HLM (Fig. 1). The first two components, awareness and knowledge, encompass the understanding acquired by individuals about lifespan, healthspan, and HLM. The third component reflects individual factors underlying motivations towards healthy longevity. These factors include personality, current behaviours, personal values and beliefs, and health-related perceptions. The components are presented below, with the scientific rationale to include each in the HELO framework.

#### Awareness of and knowledge about healthy longevity

Low health literacy can arise from either a lack of awareness or knowledge [18, 19]. It is associated with adverse health outcomes such as hospitalisations, overall poor health status, and mortality [20, 21], decreased use of healthcare services such as health screenings and vaccination [20, 21], and suboptimal health behaviours like sedentariness and a poor diet [20] (for a review see [22]). In contrast, high health knowledge is associated with the adoption of health-promoting behaviours and attitudes in older adults [23]. Nevertheless, awareness and knowledge do not always translate into optimal health behaviours. A good example is unhealthy food consumption, where individuals persist in poor eating habits despite having knowledge and awareness of the associated health risks [24, 25]. Hence, whereas awareness and knowledge may form a foundation for attitudes and behaviours about healthy longevity, they are unlikely the sole determinants.

#### Factors underlying motivations towards healthy longevity

A complex set of extrinsic and intrinsic factors may help to explain why certain people engage in certain behaviours [26]. Four sub-components of factors underlying motivations explaining views towards healthy longevity are presented.

i. Personality is the enduring set of behavioural and emotional dispositions influencing how an individual adjusts to their environment [27]. Amongst the big five personality types, conscientiousness reflects responsibility, diligence, and orderliness, and is the strongest and most stable predictor of longevity [28–30]. Personality can also influence attitudes towards healthy and unhealthy lifestyles. Extraversion, which reflects sociability, is associated with healthy practices, while neuroticism, which reflects negative emotionality, is associated with unhealthy ones [31]. However, moderate levels of certain neuroticism aspects, such as trait anxiety, may have health-protective effects [28, 32], suggesting that personality could be examined to better tailor interventions to the individual’s strengths and weaknesses.
ii. Current behaviours are often good predictors of future behaviours. For instance, individuals who engage in unhealthy behaviours, like smoking, alcohol consumption, and unhealthy dietary choices, may be more inclined to engage in other unhealthy behaviours in the future [33]. The HELO framework considers behaviours directly related to HLM, such as using HLM products or services, as well as lifestyle choices and habits more broadly associated with longevity. These associated behaviours include smoking and alcohol consumption [37], physical activity [38], and preventive health behaviours like going for regular health screenings or dental check-ups [34, 35]. As behaviours result from the interplay of awareness, knowledge, and other motivational factors, understanding current behaviours within the context of the other motivational sub-components can help identify where interventions would be most impactful in promoting healthy lifestyles and HLM.
iii. Personal values and beliefs are deeply ingrained principles guiding behaviours and attitudes, representing convictions about what holds central importance in life [36]. In the context of healthy longevity, personal values can be conceptualised in various ways, such as one’s religiosity or aspirations, which provide meaning and direction in life [37, 38]. As values and priorities may conflict as pursuing long-term health can sometimes require forgoing immediate gratification, the expectancy-value motivation model [39] may be a helpful framework for understanding health-related behaviours. That model, which has been extensively used in education research to devise learning-promoting interventions, states that behaviour is influenced by a subjective cost–benefit analysis [40]. In short, individuals consider the likelihood that an action will achieve a valued outcome. The more they can expect such an outcome, the more they will be motivated to perform that action. This model may aid in understanding individual values and priorities regarding healthy longevity and the trade-off individuals make between long-term health gains and short-term inconvenience.
iv. Health-related perceptions may influence longevity-related motivations. Ageistic social norms, for instance, can be internalised by older adults and precipitate their cognitive, mental, and physical decline [41, 42], as these embodied stereotypes reduce willingness and ambitions to engage in activities, social interactions, self-improvement, and future self-care [43, 44]. Older subjective age, or the age ‘felt’ by the individual, is negatively associated with the desire for a longer lifespan [45]. Positive everyday experiences, such as high subjective social support and quality of life, have been associated with stronger motivations to remain healthy longer [46, 47]. Additionally, perceptions of innovative approaches to health, such as HLM clinics and consumer products, could be formed based on individual’s perceived risks and benefits of engagement in healthy longevity [48, 49]. Similarly, the perception of descriptive health norms, indicating how often one believes their peers engage in a specific behaviour, can influence the behaviour’s adoption [50]. Understanding both positive and negative public perceptions related to healthy longevity and HLM is therefore an important step in finding the right tone and content in their promotion.

### HELO survey

The HELO survey incorporates a combination of validated questionnaire items and newly developed items, which operationalise the components and sub-components of the HELO framework. The HELO survey’s outline, assessment objectives, corresponding questionnaire and sources (if applicable), and any item modifications are summarised in Table 1.

**Table 1:** Outline of the HEalthy LOngevity (HELO) survey exploring public awareness, knowledge, and factors underlying motivations about lifespan, healthspan, and Healthy Longevity Medicine

Component domain/section	Assessment’s focus	Name of scale [ref]	Items design/modification and reason of change [ref]
Knowledge			
Lifespan	Lexical understanding of lifespan (total years lived)	New items	Expert opinion and literature [3]
Healthspan	Lexical understanding of healthspan (years in good health)	New items	Expert opinion and literature [3]
Biological age	Lexical understanding of biological age (age based on the health of cells in the body)	New items	Expert opinion and literature [51]
Healthy Longevity Medicine	Lexical understanding of Healthy Longevity Medicine (medical practice optimising how people age)	New items	Expert opinion and literature [52]
Awareness			
Healthy Longevity Medicine	Opinions on the effectiveness of Healthy Longevity Medicine	New items	Expert opinion
Motivation			
Personality			
Five Factor Model	Personality dimensions: Openness to experience, Conscientiousness, Extraversion, Agreeableness, and Neuroticism	Big Five Inventory—10 items [BFI-10] [53]	Item 5: replaced ‘has few’ to ‘does not have many’ Reason: feedback from face validation
Current behaviours			
Use of health services	Health services and biological tests used in the past year	Standard	NA
Smoking	Smoking frequency, quantity, and history	Smoking and Smoking History Questionnaire [54]	Merged questions for past/current smokers Item 2: shorten options to ‘Less than ‘1’, ‘1 to 20 (one pack)’, ‘20 or over’, and ‘Refuse to answer’ Item 3: amended options to ‘Less than 1 year’, ‘1 to 5 years’, ‘6 to 10 years’, ‘11 years or more’, and ‘refuse to answer’ Reason: expert opinion
Alcohol consumption	Alcoholic consumption frequency per week	Standard	NA
Physical activity	Physical activity frequency per week	[55]	No change
Health activities	Healthy lifestyle habits related to nutrition, maintenance of physical health, and health monitoring	Good Health Practices Scale [56]	Item 6: added ‘unhealthy’ to describe food and replaced ‘coffee’ with ‘sweetened drinks’ Item 15: replaced ‘shots’ with ‘vaccinations’ Item 17: added this new item on the use of wearable devices Reason: expert opinion
Personal values and beliefs			
Religiosity	Extent of religious belief	[57]	Item modified to include both religious and spiritual viewpoints Reason: expert opinion
Aspirations	Achievements of life goals related to wealth, image, personal growth, community	Aspiration Index [58]	Included two items from each of five out of seven original subscales: *wealth*, *image*, *personal growth*, *community*, *health* Response option: changed from 7- to 3-point Likert scale Reason: expert opinion
Expectancy-value-cost	Expectancy (the probability of achieving the outcome), value (the benefits of the outcome), and costs (the price of achieving the outcome) of maintaining a healthy lifestyle	Expectancy-Value-Cost Scale [59]	Included two items from each of the three original subscales All items: modified items from math/science class- to health living-focused Reason: expert opinion
Preferences about own lifespan and healthspan	Desired years of lifespan and healthspan and willingness to receive interventions for lifespan only, healthspan only, or both	[11]	Item 2: added this new item on the expected healthspan Item 3: added this new item on the perceived average lifespan Item 4: modified to merge physical and mental health Reason: expert opinion
	Monetary investment range for long-term health	New item	Expert opinion
Health-related perception			
Ageism	Perceived age-related prejudice	WHO Ageism Against Older Adults Scale–5 items [60]	No change
Subjective age	Perceived age based on how one feels	[61]	No change
Perceived social support	Perceived assistance from social networks	Perceived Social Support Questionnaire [F-SozU] [62]	Included two of the original six items Reason: expert opinion
Health-related quality of life	Perceived health-related quality of life based on mobility, self-care, usual activities, pain/discomfort, and anxiety/depression	EuroQol-5D [EQ-5D] [63]	No change
Perceived risk-benefits/secondary risk theory	Perceived risks and benefits of using healthy longevity interventions	New items	Expert opinion
Descriptive health norms	Perceived peer expectations around health-promoting behaviours	Subjective and Descriptive Norms [64]	All items: modified from ‘exercise’ to ‘lead a healthy lifestyle’ Reason: expert opinions
Socio-demographic and medical characteristics	Socio-demographic (e.g. age, sex, socioeconomic status) and medical (e.g. height, weight, diagnosis of any chronic conditions) characteristics	Standard items	NA

## Discussion

The HELO framework builds on previous population surveys, such as the McKinsey Health Institute’s global survey and the Hevolution’s global healthspan report [12, 13]. Those surveys showed the complexity of public perceptions around health and healthspan, emphasising the need for a comprehensive strategy for health promotion. By adopting an interdisciplinary approach, the HELO framework and survey aim to advance this initiative by offering new theorisation and conceptualisation of how awareness, knowledge, and factors underlying motivations shape views towards healthy longevity.

The HELO framework has several important implications for public health and policy. First, its knowledge and awareness components help identify gaps in public understanding related to healthy lifestyles and behaviours, which can inform and develop more targeted educational campaigns. These campaigns can address misconceptions, enhance health literacy, and promote healthy habits among the public. Second, the factors underlying motivations enable the identification of specific populations with views that either support or avoid healthy longevity, enabling the development of tailored strategies to reach and engage populations with lower levels of motivation towards health-promoting behaviours. This is particularly relevant for designing public health behavioural interventions and for communicating these interventions to the public to foster behavioural changes in groups that are disengaged and at risk of poorer health outcomes. Additionally, as the HELO survey will be deployed across multiple countries, cultures, and populations, the HELO initiatives offer a unique opportunity to compare national, cultural, or language-specific trends in public views and behaviours, which can be contrasted with global patterns. These insights can be leveraged to design culturally sensitive public health interventions that consider regional differences while contributing to a more comprehensive global understanding of healthy longevity. Ultimately, the HELO framework’s ability to identify and address knowledge gaps, motivational barriers, and cultural nuances holds significant potential for shaping effective public health strategies and policies aimed at improving population health and healthspan globally.

One key strength of the HELO framework is its integration of evidence-based concepts from diverse disciplines, including medicine, geroscience, social and behavioural psychology, and public health. The use of validated scales supporting the framework’s structured focus on awareness, knowledge, and factors underlying motivations allows for a more nuanced exploration of the factors shaping public perspectives, which, however, may be subjected to local contexts. Depending on the variability of findings, further iterations of the survey may be needed to address concepts, that, while not significant in Singapore, could be relevant in other regions. Nevertheless, the HELO initiative offers an evidence-based background for developing and implementing effective interventions and health policies, while also serving as a baseline to monitor changes in public perspectives over time.

As the next steps, the HELO survey will be contrasted with the findings from focus group discussions conducted with diverse members of the Singaporean public. This qualitative validation step will ensure that the concepts included in the survey capture the target population’s perspectives on the topics of lifespan, healthspan, and HLM. The HELO survey will then be deployed to the general public in Singapore. The established international HELO consortium will then further adapt the survey to general populations of other countries as well as particular subpopulations of interest for consortium members, such as healthcare professionals or intergenerational families, to explore cross-cultural perspectives.

## Conclusion

The HELO framework includes public awareness, knowledge, and motivations, and serves as a conceptual foundation for a population survey intended to identify and monitor factors shaping public attitudes towards lifespan, healthspan, and HLM in different nations and populations of interest. The understanding obtained from the survey is expected to advance the development and implementation of effective, targeted interventions to improve knowledge and shape policies optimising population health and healthspan.
